# Characterisation of Wood Particles Used in the Particleboard Production as a Function of Their Moisture Content

**DOI:** 10.3390/ma15010048

**Published:** 2021-12-22

**Authors:** Dorota Dukarska, Tomasz Rogoziński, Petar Antov, Lubos Kristak, Jakub Kmieciak

**Affiliations:** 1 Department of Mechanical Wood Technology, Poznań University of Life Sciences, 60-627 Poznań, Poland; j.kmieciak24@gmail.com; 2 Department of Furniture Design, Poznań University of Life Sciences, 60-627 Poznań, Poland; tomasz.rogozinski@up.poznan.pl; 3 Faculty of Forest Industry, University of Forestry, 1797 Sofia, Bulgaria; p.antov@ltu.bg; 4 Faculty of Wood Sciences and Technology, Technical University in Zvolen, 960 01 Zvolen, Slovakia; kristak@tuzvo.sk

**Keywords:** wood particles, moisture content, angle of repose, slippery angle of repose, poured bulk density, tapped bulk density

## Abstract

The properties of particleboards and the course of their manufacturing process depend on the characteristics of wood particles, their degree of fineness, geometry, and moisture content. This research work aims to investigate the physical properties of wood particles used in the particleboard production in dependence on their moisture content. Two types of particles currently used in the production of three-layer particleboards, i.e., microparticles (MP) for the outer layers of particleboards and particles for the core layers (PCL), were used in the study. The particles with a moisture content of 0.55%, 3.5%, 7%, 10%, 15%, and 20% were tested for their poured bulk density ($\rho_{p}$), tapped bulk density ($\rho_{t}$), compression ratio (*k*), angle of repose ($\alpha_{R}$), and slippery angle of repose ($\alpha_{s}$). It was found that irrespective of the fineness of the particles, an increase in their moisture content caused an increase in the angle of repose and slippery angle of repose and an increase in poured and tapped bulk density, while for PCL, the biggest changes in bulk density occurred in the range up to 15% of moisture content, and for MP in the range above 7% of moisture content, respectively. An increase in the moisture content of PCL in the range studied results in a significant increase in the compression ratio from 47.1% to 66.7%. The compression ratio of MP increases only up to 15% of their moisture content—a change of value from 47.1% to 58.7%.

## 1. Introduction

The physicomechanical properties of particleboards, except their technological parameters, depend significantly on the characteristics of the raw materials used in their production [1]. These are the type and amount of the adhesive system used for their bonding, the type of the raw material, its degree of fragmentation and geometry (size and shape), and moisture content [2,3,4,5,6,7,8,9,10,11,12]. The right choice of the moisture content of the particles, independently of their fineness degree, is also necessary for assuring the correct industrial process of particleboards. The excessive moisture content of particles may cause delamination of the particleboards during their pressing [13]. In turn, overly dry particles increase the risk of fires during drying and also contribute to the formation of wood dust, which disturbs the process of particles bonding or mat densification during hot pressing. One of the stages of wood particles preparation for particleboards production, independently of the material they originate from and bonding agents used, is the energy-consuming drying operation. Therefore, the possibility of producing boards from particles with higher moisture content is an opportunity to optimise energy consumption in production plants, e.g., by reducing the work of dryers or selecting appropriate drying conditions and thereby reducing the production costs [14,15]. Furthermore, the aspect of wood particles’ moisture content also seems to be of interest due to the trend of using isocyanate adhesives in the production process. Conventional thermosetting aminoplastic resins show deteriorated water resistance, which results from the hydrolysis of the methylene bridges [16,17]. However, polymeric 4,4′-diphenylmethane diisocyanate (pMDI) adhesives show high reactivity and the ability to a chemical reaction with wood components and the water they contain. It profitably influences the bonding quality of the particles and thereby the physicomechanical properties of particleboards [4,18,19,20]. These properties were studied by Jiang and Lu [21] for producing boards meeting the requirements of the EN 312 standard [22] prescribed for P2 type boards from particles with a moisture content of 25%, bonded with melamine–urea–formaldehyde resin (MUF) modified with different additional proportions of polyurethane prepolymer. As studies showed, the use of pure MUF resin for bonding particles with moisture content above 20% leads to the blowout of particleboards [13].

While preparing particles for the production of particleboards, it should also be considered that the moisture content of the mat increases together with the moisture content of the particles, which influences the parameters of the bonding process and the quality of particles bonding and, as a result, the properties of the finished boards. The moisture content of the mat is one of the most important factors influencing the heat transfer in the mat [23]. The rate of heat penetration in the mat determines the pressing time, which is critical for the efficiency of the production process [24]. It influences the increase in the temperature in the mat, which determines the cure rate of the adhesive resin. The combination of high temperature, moisture content, and time may cause an excessive increase in the vapour pressure in the mat and trigger an explosion when the press is opened [23,25]. However, the technological problems connected with the excessive moisture content of wood particles and vapour pressure during pressing can be effectively solved. Murayama et al. [26] investigated the temperature variability and vapour pressure during pressing of the particleboards and concluded that this problem can be solved by choosing an optimal moisture content of board layers. The increased moisture content of the face-layer and the lower face-layer thickness was expected to reduce the time of reaching the required temperature in the hot-pressing process. The usage of air-injection during the pressing of boards can also be a solution [19]. In the developed method, the air-injection press, which has holes punched in the heating plates, injects high-pressure air into the board through the holes of one plate and releases the air through the holes of the other plate. The advantage of this way of board pressing is allowing for reducing the pressing time required for manufacturing boards from high-moisture-content particles. Unfortunately, the air-injection press could not improve the properties of the particleboards [13].

As it results from the above considerations, the issue of the influence of particle moisture on the properties of finished particleboards and the course of their production process is practically well known. However, an often overlooked issue is the effect of particle moisture content on their physical properties such as poured bulk density, tapped bulk density, angle of repose, and slippery angle of repose. These properties are relevant because they influence the method and conditions of their storage and transport. They also influence the course of the production process, including the operation of transport and dosing devices and the choice of proper technological parameters during the bonding and pressing of particles, and consequently the properties of finished particleboards. Moisture content affects characteristics of bulk solids including wood and lignocellulosic particles such as particle-size distribution and bulk density [10,27,28]. The range of changes in these properties depending on the moisture content of the particles should be known in order to be able to assess its possible influence on the course of the production process and the properties of particleboards. Considering the above aspects, this work aimed to evaluate the physical properties of wood particles commonly used in the production process of three-layer particleboards depending on their moisture content.

## 2. Materials and Methods

### 2.1. Materials

**Figure 1:**
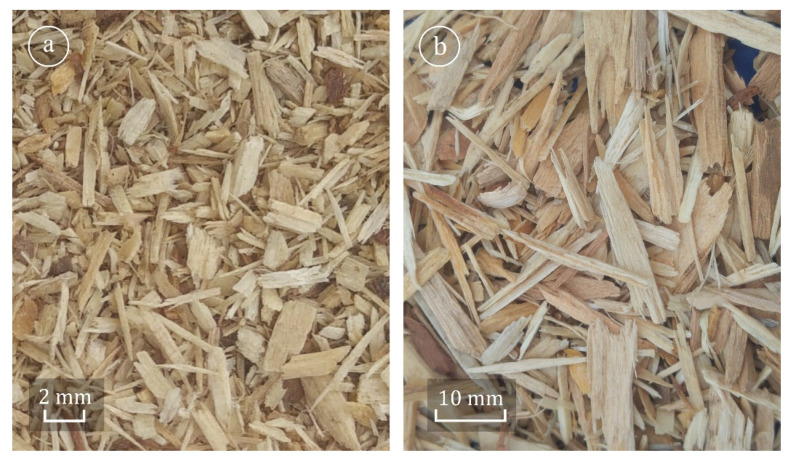
Images of wood particles: (**a**) microparticles (MP), (**b**) particles of the core layer particleboards (PCL).

Two types of wood particles used in the production of three-layer particleboards were used in the study, i.e., so-called microparticles intended for outer layers of particleboards and particles of the core layer (Figure 1).

These particles have been produced under industrial conditions mainly from middle- and small-sized softwoods and selected low-density hardwood species. The raw material was also residues from the sawmilling industry in the form of shavings, sawdust, and chips. The initial moisture content of wood particles, determined by the drying–weighing method, was 7 ± 0.5%. To achieve the intended goal, the particles were submitted to drying or moisturising to the moisture content of 0.55%, 3.5%, 7%; 10%, 15%, and 20% ± 0.5%. In effect, the moisturising of different types of particles can be conducted directly or indirectly by increasing the moisture content of the environment [29]. In the present study, the first method was used i.e., wetting of the particles by spraying them with an appropriate amount of water and in order to homogenise the moisture content in the whole mass by seasoning for a period of 72 h. The moisture analyser MA R. 50 (Radom, Radwag, Poland) was used for the control of the moisture content of the particles. It determines the moisture content of the material on the basis of weight losses of the tested sample during its heating at a determined drying temperature (105 °C was used in the study). For preliminary characterisation of the raw materials used in this study, their fractional composition was determined for particles with 0.55% moisture content and additionally for particles with 20% moisture content. In the case of PCL particles, the fractional composition was determined based on sieve analysis, with the use of flat sieves made of mesh with square perforations of 6.3, 5.0, 4.0, 2.5, 1.6, 1.0, and 0.5 mm. In turn, for MP particles, sieves with mesh sizes of 3.15, 1.25, 1.0, 0.63, 0.4, and 0.315 mm were used. For PCL particles, due to the greater differences in the shape and size of individual particles, the additional dimensional analysis was carried out, for which 250 particles of the predominant fraction (from a 2.5 mm mesh sieve) with the moisture content of 0.55% were drawn. Their length, width, and thickness were determined. This allowed the estimation of the basic shape factors of this type of particles, i.e., the degree of slenderness ($\lambda_{s}$), flatness (*ψ*), width coefficient (*m*), and specific surface area ($F_{w}$) estimated according to the equations shown below [10]:

(1) $$\lambda_{s}=\frac{l}{h}\;$$

(2) $$\psi =\frac{b}{h}\;$$

(3) $$m=\frac{l}{b}\;$$

(4) $$F_{w}=\frac{0.002n}{w}\left(lh+lb+bh\right)$$

where *l*—mean length of wood particles (mm), *h*—mean thickness of wood particles (mm), *b*—mean width of wood particles (mm), *w*—mean weight of dry wood particles (g), *n*—number of wood particles selected for analysis, 0.002—coefficient taking into consideration the fact that wood particles have two surfaces and a unit converter from mm to m.

Such analysis in the case of particles with a diverse geometry (Figure 1) is justified because of the fact that the biomass particles are mostly inhomogeneous in terms of size and shape [30]. As a result, two particles going through the same sieve with the same mesh size may differ in shape. Therefore, the information obtained from the sieving process may not fully reflect the geometry of the biomass particles with such an irregular shape [31]. The parameters characterising the geometry of the PCL particles are presented in Table 1.

Subsequently, the prepared material was tested for the influence of the moisture content of the particles on their poured bulk density ($\rho_{p}$), tapped bulk density ($\rho_{t}$), compression ratio (*k*), angle of repose ($\alpha_{R}$), and slippery angle of repose ($\alpha_{s}$).

### 2.2. Poured Bulk Density and Tapped Bulk Density of Wood Particles

The first parameter, the poured bulk density, was expressed as the ratio of the weight of loosely poured wood particles to their volume. To determine the effect of the moisture content of the tested particles on their tapped bulk density, particles loosely poured into a pot equipped with a volume scale were densified on a lab electromagnetic vibratory sieve shaker AS200 (Retsch GmbH, Haan, Germany) in the time of 10 min and with the vibration amplitude of 2 mm. The tapped bulk density was expressed as the ratio of the weight of poured wood particles to their volume recorded after the tapping. Based on the obtained results of the poured bulk density and tapped bulk density, the compression ratio (*k*) of the tested particles was determined, depending on their moisture content according to the equation [10]:

(5) $$k=\left(\frac{\rho_{t}}{\;\rho_{p}}\cdot 100\right)-100$$

where *k*—compression ratio (%), $\rho_{p}$—poured bulk density (kg/m^3^), and $\rho_{t}$—tapped bulk density (kg/m^3^).

The average values of poured bulk density and tapped bulk density for each tested variant were determined based on five unitary measurements.

### 2.3. Angle of Repose and Slippery Angle of Repose of Wood Particles

**Figure 2:**
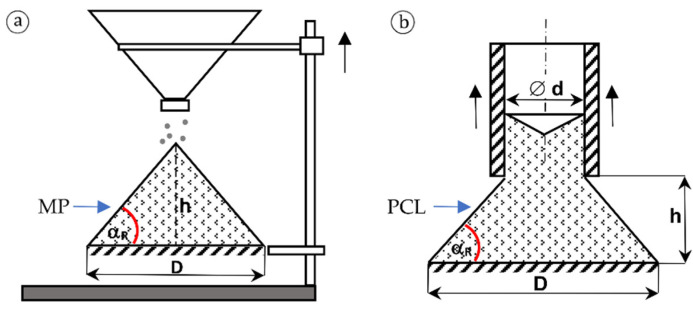
Scheme of the device measuring the angle of repose of: (**a**) microparticles, (**b**) particles of the core layer of particleboards.

In general, the angle of repose is defined as the angle between the slant height and the base of a cone created during the loose falling of bulk material at right angles to the ground. However, due to the differences in the fineness and therefore the size of the tested particles, other procedures were used to determine the influence of the moisture content on the angle of repose of the particles. In the case of microparticles, the analysis of the angle of repose (according to PN-74 Z-04002.07 standard [32]) was based on pouring them in a steel discharge hopper with a calibrated hole with the diameter of 22 mm, which was attached to the base with a gear train (Figure 2a). Next, the hopper was being lifted with a linear movement to the moment of pouring of the microparticles on a plate with the diameter of 120 mm. After piling up a stable cone, its height was measured. In contrast, in the case of PCL-type particles, the angle of repose was determined by pouring them into a cylinder with the diameter of 120 mm and the height of 100 mm, and then by lifting it, a cone from the particles was formed whose height was decoded from the millimetre scale attached to the base of the device (Figure 2b) [33].

The angle of repose for both types of wood particles tested was estimated based on the equation:

(6) $$tg\;\alpha_{R}=\frac{2h}{D-d}$$

where $\alpha_{R}$—angle of repose [°], *h*—cone height [mm], and *D*—cone base diameter [mm]. In the case of MP (Figure 2a), the value of *d* = 0.

**Figure 3:**
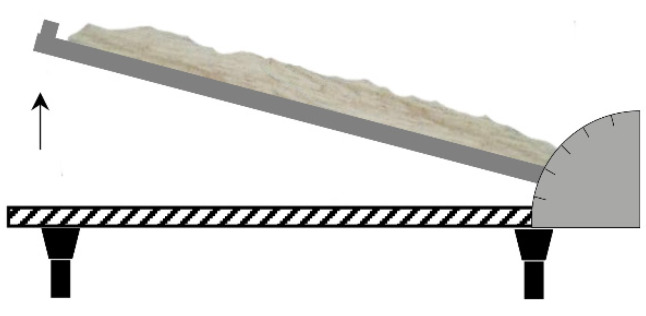
Scheme of the device measuring the slippery angle of repose of tested particles.

The analysis of the slippery angle of repose was based on evenly pouring particles with the weight of about 200 g (MP and PCL) on a levelled and flat surface of the measuring device and then by lifting its side edge, finding the minimal rake angle that causes pouring of the layer of material. The slippery angle of repose value was directly decoded from a protractor pitch attached to the base of the device (Figure 3).

The mean values of angle of repose and slippery angle of repose were determined based on five unitary measurements.

### 2.4. Statistical Analysis

The obtained results of the tests selected for wood particles were statistically analysed using the STATISTICA v.13.1 software (StatSoft Inc., Tulsa, OK, USA). The mean values of the determined parameters were compared in the one-way analysis of variance—Tukey’s post hoc test, in which homogeneous groups of mean values for each parameter were identified for *p* = 0.05. In the case of the fractional composition, a two-factor analysis of variance was used, assuming the size of the fraction and wood particle moisture as a qualitative factor.

## 3. Results

### 3.1. Fractional Composition of Wood Particles

**Figure 4:**
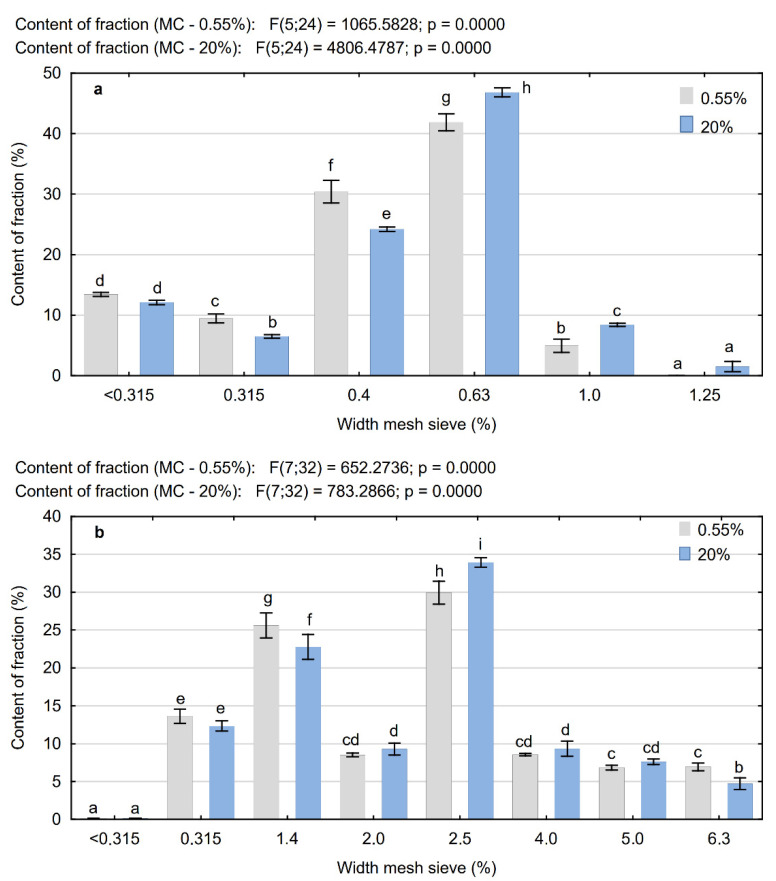
Fractional composition of microparticles (**a**) and particles of the core layer of particleboards (**b**) depending on their moisture content (a, b, c …—homogeneous groups as determined by the Tukey test).

The wood raw materials used in the study, independently of the degree of their fineness, posed a mixture of particles with diverse shapes and sizes. It is proclaimed by images of particles (Figure 1), the estimated coefficient of PCL shape (Table 1), and their fractional compositions (Figure 4), which were determined for dry particles (0.55%) and additionally for particles with the moisture content of 20%. Based on these diagrams, it can be concluded that in the case of MP independently of their moisture content, the particles of fractions 0.63 mm and 0.4 mm had the largest weight share. At a moisture content of 0.55%, the share of 0.63 mm fraction was 41.9%, and at a moisture content of 20%, it was slightly more, i.e., about 46.8%. In contrast, for the MP of fraction 0.4 mm, the share is greater by approximately 25%, which was observed for the moisture content of 0.55% in relation to the moisture content of 20% (respectively 30.4% and 24.2%). A similar dependence can be observed in the case of MP of a finer fraction, i.e., <0.4 mm. In contrast, the tests of the PCL particles showed that the particles of fractions 2.5 and 1.4 mm had the biggest share in the whole mixture. The content of dry particles (i.e., with the moisture content of 0.55%) of fraction 2.5 mm remained on the level of about 30%, whereas dry particles of fraction 1.4 mm were on the level of about 25.6%. Analogically to MP, for the particles of this type, it was also observed that the increase in their moisture content caused a significant differentiation of their fractional composition. In general, it can be stated that the increase in the moisture content of particles resulted in a decreased share of the finer fractions (smaller than the predominant fraction) and increased share of the larger fractions. The observed differences were statistically significant, which was confirmed by the two-factor post hoc analysis, which in the case of MP of fractions 0.315–1.0 mm allowed for distinguishing six homogenous groups. In the case of PCL, statistically the greatest differences were observed for the fractions 1.4, 2.5, and 6.3 mm. The observed changes in the fractional composition of the tested particles can be explained by the fact that at higher moisture content of the particles, an insufficient separation of MP occurs due to their adhesion to larger particles. Finer particles show the ability to agglomerate with the larger ones, which escalated with the increase in their moisture content [34].

### 3.2. Poured Bulk Density and Tapped Bulk Density of Wood Particles

**Figure 5:**
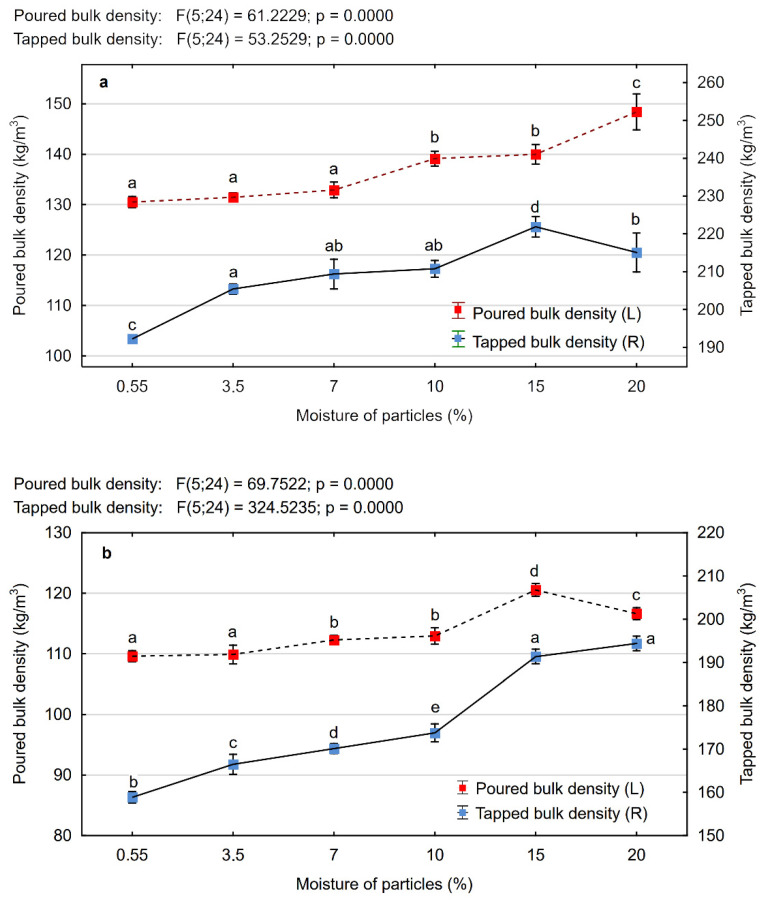
Poured bulk density and the tapped bulk density of microparticles (**a**) and wood particles of the core layer of particleboard (**b**) depending on their moisture content (a, b, c …—homogeneous groups as determined by the Tukey test).

The poured bulk density of particles of different biomass types are dependent on their shape, size, the way of their forming in the mass, and the friction between the particles [35,36]. In the case of material such as wood particles, wood chips, or wood dust, the poured bulk density also depends on the absolute density of the wood itself [37]. Figure 5 presents the results of tests on the influence of particle moisture content on the formation of their poured bulk density and tapped bulk density. It was determined that in the case of MP particles, the increase in moisture content from 0.55% to 7% did not cause statistically significant differences in the values of the poured bulk density. The observed mean values $\rho_{p}$ in Tukey’s test were classified in the same homogenous group (a). The further increase in the moisture content resulted in a gradual increase in the poured bulk density.

The MP-type particles with a moisture content of 20% showed the $\rho_{p}$ value higher by about 14% than the ones with the lowest moisture content, whereby the highest increase in density occurred by changing the moisture content from 15% to 20%. In the case of PCL, the increase in poured bulk density was observed just by 9% but to the moisture content of 15%. Regarding the PCL particles with a moisture content of 20%, a small but statistically significant decrease $\rho_{p}$ was observed. Previous studies on the influence of particle moisture content of different types of biomass (almond nut, sunflower seed, flaxseed, straw) on their physical properties showed that this relationship can develop in different ways [2,10,38,39,40]. For example, Dukarska et al. [10] and Aviara et al. [40] proved that the bulk density of seeds (*Moringa oleifera)* or straw particles of selected grain species increased with respect to an increase in moisture content. In contrast, other researchers such as Littelield et al. [41] showed that the bulk density of pecan shells decreased with an increase in moisture content. According to the authors, the decrease in the poured bulk density of the particles is caused by the increase in their size under the influence of the moisture content and thereby their volume, which was following on faster than the weight increase as a result of increasing the moisture content. As expected, along with the increase in the moisture content of particles, their tapped bulk density also increased. Considering the statistical analysis, the influence of the moisture content of particles on their tapped bulk density was greater than in the case of poured bulk density. It can be observed especially with regard to the PCL particles. Analysing the results obtained for MP, it can be stated that this density increased more with the increase in the moisture content from 0.55 to 3.5% and from 10% to 15%. However, the increase in the moisture content of MP from 0.55% to 20% caused the increase in their tapped bulk density by about 12% and in the case of PCL by about 22%. It results from the fact that the higher moisture content of MP (similar to wood dust) contributes to an increase in their volume, which causes the decrease in free space around them and the increase in consistency of the whole mass of particles [10,27,28]. In practice, the raw material with higher moisture content, independently of their degree of fineness, requires an increase in the volume essential to their storage or transport [41]. Moreover, it can be concluded that at higher moisture content, the PCL particles are more susceptible than MP to compaction by vibration, which can cause some difficulties in the technological process of producing particleboards and during their transport.

Comparing the results from Figure 5, it was also established that in the tested range of the moisture content, MP were marked by higher poured and tapped bulk density than the particles used for the core layers of particleboards. The literature shows that fine particles (such as MP characterised by their small size) are better at filling empty spaces during their pouring than larger particles. As the sizes of the particles increase, larger particles cannot sufficiently fill the empty spaces during tapping, which causes the decrease in their tapped bulk density [41]. This corresponds with the works of other authors who, studying the effect of particle size of different types of biomass, also observed that the value $\rho_{t}$ increases with decreasing particle size of cereal straw, corn straw, switchgrass, and nutshells [35,41,42]. As demonstrated by Dukarska et al. [10], in the case of cereal straw, stem morphology is also an important factor that is related to the size and geometry of the particles, in particular, their thickness and degree of slenderness.

### 3.3. Compression Ratio

**Figure 6:**
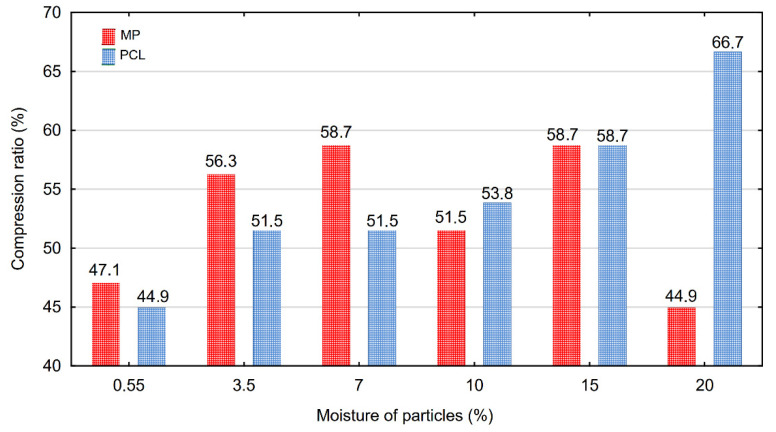
The compression ratio of wood particles depending on their moisture content.

The changes in poured and tapped bulk density caused by the changes in the moisture content of the tested wood particles are reflected also in their compression ratio. A graphical representation of the compression ratio of wood particles depending on their moisture content is presented in Figure 6. It can be stated that in the case of PCL, along with the increase in their moisture content from 0.55% to 20%, a gradual and significant increase in the value of this parameter by about 48% was observed. The increase in the degree of fineness of the wood raw material to MP caused the decrease in vulnerability to its compressibility. It was observed that the increase in their moisture content resulted in the increase in the compression ratio from 44.9% to 58.7%, so by about 25% in comparison to the dry particles, however just in the moisture content range to 15%. By 20% moisture content of the MP, the decrease in its moisture content was set down to the level, which had been set down for dry particles. This phenomenon probably results from the small sizes of MP and their significant compaction in the whole mass. Moreover, increasing their moisture content resulted in reduced free spaces between particles and increased their adhesion to each other. When a particulate system becomes damp, the cohesion increases due to the creation of the liquid bridge bonds between the particles. The system remains stable until the moisture content is too high for strong bridges. When the particulate system becomes more and more wet, the material reaches the state of a slurry. During drying out the dump particulate system, the solid bonds and bridges between the particles can be formed. As a result of this, the material will become cohesive again. Changes in moisture content and resulting changes in other properties of a particulate system can cause serious problems in handling particles in an industrial installation [43]. In addition, biological materials (which may also include wood particles) become softer; thus, deformation is greater with an increase in moisture content [41]. In contrast to MP, increasing the moisture content of PCL chips from 15% to 20% results in a further increase in their compression ratio up to 66.7%. This might be attributed to the changes in the dimensions of the particles on account of their swell influenced by the increasing moisture content. Along with increasing dimensions of the particles, the volume of the space between them in a layer also increased and can be filled with the particles during tapping.

### 3.4. Angle of Repose and Slippery Angle of Repose of Wood Particles

**Figure 7:**
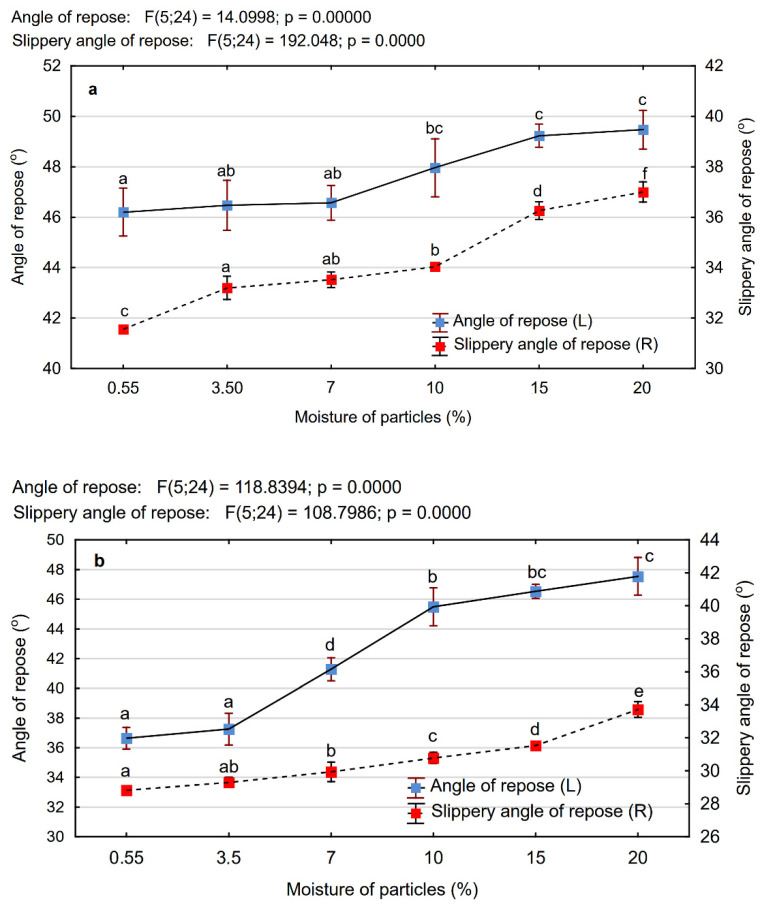
The angle of repose and the slippery angle of repose of microparticles (**a**) and particles of the core layer of particleboard (**b**) depending on their moisture content (a, b, c …—homogeneous groups as determined by the Tukey test).

The angle of repose and slippery angle of repose are important physical parameters characterising wood particles. Graphical representation of the angle of repose and the slippery angle of repose of MP and PCL depending on their moisture content is presented analytically in Figure 7. Independently of the fineness of the particles, along with increasing their moisture content, the angle of repose and slippery angle of repose also increased. However, it can be observed that the biggest changes in values were recorded for the angle of repose of PCL particles. In this case, the increased moisture content from 0.55% to 20% resulted in increased $\alpha_{R}$ from 36.6° to 47.6°, i.e., by about 30%, whereby, as the variance analysis showed, statistically significant differences were set down for the moisture content higher than 3.5%. This is evidenced by the results of the analysis of variance, which identified five different homogeneous groups for each PCL moisture content above this value. Increasing the moisture content had much less effect on the angle of repose of the MP, for which the maximum value of $\alpha_{R}$ totals just 7%. Markedly, the greatest differences between MP and PCL particles were observed in the range of lower moisture content (up to 7%). This is an important observation in view of the fact that in the industrial practice, depending on the technology, the moisture content of the particles for the outer layers of the three-layer particleboards varies from 2% to 8% and in the case of the core layers, it varies from 1% to 6%. The results of the multivariate significance tests ANOVA presented in Table 2 are a confirmation that the lack of interactions between the tested effects (the type and moisture content of particles) were in the range of the higher moisture content, over 10% (*p* > 0.05). The results obtained for the angle of repose and the slippery angle of repose can be explained by the differences in their sizes and shape as well as by their consistency and looseness. The physical properties of wood particles, e.g., the size, shape, or roughness of the surface significantly affect the looseness of the particles of different types of biomass [30,31,36]. According to the subject literature, the increased particle size is connected with the decrease in their tenacity and therefore the value of the angle of repose [30]. In turn, fine particles, which can include wood MP, have a higher specific surface area, which increases the contact and cohesiveness among the particles, which may cause difficulties in their flow in technological conditions [31].

With regard to the slippery angle of repose, no significant differences were determined. Independently from the degree of fineness of the particles the increase in the value $\alpha_{s}$ was on the same level, i.e., 17% on average. The increase in the slippery angles of repose along with the increased moisture content of particles may indicate an increase in their traction and thereby restriction of their mobility. Comparing the data obtained for both tested types of wood particles, it can be concluded that the values of MP’s slippery angles of repose were greater than the PCL particles of particleboards.

## 4. Conclusions

Based on the conducted research, the influence of the moisture content variation on the physical properties of PCL and MP was determined. It was demonstrated that the increase in the moisture content of the particles resulted in increased dimensions of wood particles, independently of their degree of fineness, and increased values of the slippery angle of repose and the angle of repose. Furthermore, the increased moisture content of wood particles led to enhanced tapped bulk density for both types of studied wood particles for the production of three-layer particleboards. Regarding the poured bulk density, increased values were determined at 15% moisture content and over 7% moisture content for the PCL particles and MP, respectively. The PCL particle compression ratio was also increased. With regard to the MP particles, this increase was determined only at moisture content values up to 15%. The results of the present study can be utilised in the industrial practice of the wood-based panel industry to optimise the technological parameters and production costs related to particleboard manufacturing. 

## Figures and Tables

**Table 1:** Characteristics of PCL particles of the predominant fraction.

Parameter	Value
Average dimensions (mm):	
length (*l*)	19.1 * ± 6.2 **
width (*w*)	3.2 ± 1.0
thickness (*h*)	1.38 ± 0.4
Shape factors:	
$\mathrm{degree}\;\mathrm{of}\;\mathrm{slenderness}\;(\lambda_{s}$)	13.84
flatness (*ψ*)	2.32
width coefficient (*m*)	6.23

* mean value, ** standard deviation.

**Table 2:** Multivariate significance tests of the MP and PCL particle angle of repose.

Effect	F	P
Moisture content range: 0.55–7%		
Type of particles	F (1; 482.9) = 623.94	0.000
Particle moisture content	F (2; 17.79) = 22.99	0.000
Type of particles × particle moisture content	F (2; 14.01) = 18.11	0.000
Moisture content range: 10–20%		
Type of particles	F (1; 41.89) = 44.85	0.001
Particle moisture content	F (2; 8.13) = 8.71	0.00
Type of particles × particle moisture content	F (2; 0.4) = 0.43	0.65

## Data Availability

Not applicable.
